# Nicotine promotes neuron survival and partially protects from Parkinson’s disease by suppressing SIRT6

**DOI:** 10.1186/s40478-018-0625-y

**Published:** 2018-11-08

**Authors:** Justin W. Nicholatos, Adam B. Francisco, Carolyn A. Bender, Tiffany Yeh, Fraz J. Lugay, Jairo E. Salazar, Christin Glorioso, Sergiy Libert

**Affiliations:** 1 Department of Biomedical Sciences, Cornell University, Ithaca, NY 14853 USA; 2 Paul F. Glenn Laboratory, Department of Biology, Massachusetts Institute of Technology, Cambridge, MA 02139 USA

**Keywords:** Parkinson’s disease, SIRT6, Nicotine, Neuroprotection, Neurodegeneration, Cell death

## Abstract

Parkinson’s disease is characterized by progressive death of dopaminergic neurons, leading to motor and cognitive dysfunction. Epidemiological studies consistently show that the use of tobacco reduces the risk of Parkinson’s. We report that nicotine reduces the abundance of SIRT6 in neuronal culture and brain tissue. We find that reduction of SIRT6 is partly responsible for neuroprotection afforded by nicotine. Additionally, SIRT6 abundance is greater in Parkinson’s patient brains, and decreased in the brains of tobacco users. We also identify SNPs that promote SIRT6 expression and simultaneously associate with an increased risk of Parkinson’s. Furthermore, brain-specific SIRT6 knockout mice are protected from MPTP-induced Parkinson’s, while SIRT6 overexpressing mice develop more severe pathology. Our data suggest that SIRT6 plays a pathogenic and pro-inflammatory role in Parkinson’s and that nicotine can provide neuroprotection by accelerating its degradation. Inhibition of SIRT6 may be a promising strategy to ameliorate Parkinson’s and neurodegeneration.

**Electronic supplementary material:**

The online version of this article (10.1186/s40478-018-0625-y) contains supplementary material, which is available to authorized users.

## Introduction

Parkinson’s disease (PD) is an age-associated neurodegenerative disorder characterized by progressive death of dopaminergic (DA) neurons, leading to motor dysfunction, behavioral changes, and often dementia. No therapy exists to prevent neuronal death or halt advancement of PD [40]. Several genetic risk factors have been identified in familial cases of Parkinson’s, such as mutations in α-Synuclein, *LRRK2,* and Parkin [13], however, it is still not clear what causes the death of DA neurons at advanced age in the majority of sporadic cases, which constitute over 93% of PD [53]. Epidemiological studies have identified several factors that increase prevalence of PD, such as exposure to herbicides, certain dairy products [24], traumatic brain injury [3], or being overweight [12]. Surprisingly, in 1959 a U.S. Government-sponsored study of health among 200,000 veterans reported that smoking reduced PD deaths by 64% [15]. The negative association between tobacco use and PD and has been firmly established in over seventy independent studies [10, 15, 33, 34, 39, 57, 66]. Because tobacco smoke is a potent carcinogen, the “competing death risk” theory has been investigated and rejected [16]. Some have also suggested a reverse causation explanation, where patients are more likely to quit smoking before PD development [45]. However, the reduction of PD risk by tobacco is dependent on the duration and intensity of use [11], and second hand exposure in “never-smokers” is also protective [49], further supporting a causative link. Moreover, the tobacco component nicotine is believed to be a major mediator of neuroprotection [5]. The mechanism of tobacco and nicotine’s protective actions on PD remain unclear, but researching this phenomenon presents an opportunity to identify new therapeutic targets.

SIRT6 is a member of the sirtuin family, which comprises NAD^+^-dependent enzymes that have emerged as targets of interest for age-associated disorders, including neurodegeneration [23]. Both SIRT6 inhibitors [21, 42] and activators [19] are being developed to treat a variety of diseases, but SIRT6 has never been studied in the context of PD before. SIRT6 activity promotes apoptosis in numerous cell types [63], thus its activation is suggested to be beneficial for certain cancers [50]. However, SIRT6 activity can also promote apoptosis in non-cancer cells, including neurons [9, 43]. In fact, SIRT6 inhibition was recently demonstrated to suppress stress-induced apoptosis [14, 51] and protect from retinal neurodegeneration [67]. SIRT6 promotes production and secretion of inflammatory cytokines [4, 26, 27, 62], and chronic inflammation is thought to underlie neuronal death in PD and other neurodegenerative diseases. Tobacco smoke, a PD risk reducing factor, has been shown to decrease the abundance of SIRT6 in human lungs and in cell culture [56], while positive risk factors, such as paraquat and fatty acid overabundance both increase SIRT6 activity [18, 36]. These data suggest that SIRT6 might play a pathogenic role in PD, a topic that we investigate in this study.

SIRT6 overexpression is shown to extend longevity of mice [30], and ameliorate certain age-associated diseases in rodents [36, 50]. Based on this logic, SIRT6 is expected to protect against most age-associated diseases, including PD. However, rodents do not naturally develop PD, even at advanced age. Based on known SIRT6 functions, it is possible that SIRT6 activity has differential impact on human diseases of aging, which warrants detailed investigation of the relationship between SIRT6, neurodegeneration, and environmental risk factors for PD.

## Materials and methods

### In vitro cell culture experiments

#### Immortalized fibroblasts

Mouse embryonic fibroblasts were cultured in DMEM supplemented with 10% fetal bovine serum and penicillin/streptomycin antibiotics. The immortalized WT and SIRT6 null fibroblasts were previously generated in the laboratory of and are a generous gift from Dr. Raul Mostoslvasky.

#### Primary neuronal cultures

Primary neurons were isolated from P0 mouse pups by a standard protocol [8] modified for our study. Briefly, after dissection of neonatal brains, cortices were minced and digested in papain for 30 min at 30C^o^ temperature. After that the solution was filtered through a 100 μm filter and then fractionated in a sucrose gradient. The gradient fractions containing neurons were collected and re-suspended in Neurobasal Media, and cells were counted and plated on poly-D-lysine coated plates. The neurons were cultured in Neurobasal Media with physiological concentrations of glucose (2.5 mM) at physiological concentrations of oxygen (5%) and supplemented with bFGF and B27. Cultures were treated and analyzed 7 days after plating. Histochemical analysis performed on every batch of cells confirmed that cells were comprised of 75% neurons and 25% astrocytes. Proportions were identified by flow cytometry with the markers- NeuN and GFAP respectively (Additional file 1: Figure S5). The neuron population included tyrosine hydroxylase expressing cells confirmed by SDS-PAGE analysis. Proportion of neural cell types were not changed between WT, SIRT6 KO, and OX cultures.

To prevent mycoplasma, bacterial and fungal contamination streptomycin, penicillin, and amphotericin b were used in manufacturer specified concentrations.

#### Flow cytometry & apoptosis analysis

Primary neurons or fibroblasts were collected from wells using Trypsin digestion. Cells were washed in PBS and then suspended in 100 μL of 1X binding buffer (10 mM HEPES, 140 mM NaCl, 2.5 mM CaCl_2_, pH 7.4) with 5 μL of Annexin-V conjugate and propidium iodide (apoptotic markers). After a 15-min incubation another 400 μL of binding buffer was added and then at least 10,000 cells were analyzed using a 3 laser/ 8 color Beckton-Dickinson LSR II. A cell was considered “alive” if it was negative for both Annexin-V and PI staining. In the case of NeuN and GFAP markers, primary cultures were permeabilized with triton to allow intracellular staining.

#### Nicotine

-(−) nicotine was used for all cell culture experiments (Sigma, Cat# N3876). Fresh dilutions were made for every experiment. Diluted in culture media.

#### MG132

((R)-MG132, Cayman, Cat# 13697–1). Diluted in DMSO.

#### MK-2206 2HCl

AKT inhibitor was added 1 h before stress treatment at 1 μm concentration. Sellekchem, Cat # S1078. Diluted in DMSO.

#### Soluble TNF-RI recombinant human protein

Recombinant TNFα receptor inhibitor was added 1 h before stress treatment at 100 ng/mL. Life Technologies, Cat# PHR3015.

#### Cigarette smoke extract

Cigarette smoke was extracted by a custom vacuum device. Briefly, two 100 mm Marlboro cigarettes were burnt completely, and their smoke vacuum was collected and bubbled through 20 mL of Neurobasal Media for 1 min. This media was considered 100% cigarette smoke extract (CSE), which was later diluted and applied to cells at various concentrations.

### Human GWAS meta-analysis

The ROS-MAP cohorts are community-based cohort studies of aging in which all participants are organ donors [6]. We used 438 brains from subjects aged 67–108 years old with transcriptomic data including subjects with and without a variety of clinical diagnoses and phenotypes. Subjects were 2/3 female and predominantly Caucasian. Cohort characteristics can be found in the table below. In the original study, RNA was extracted from fresh frozen cortex sections and processed and analyzed using standard commercial RNA sequencing methods. RNA integrity was between 5.0 and 9.9, and postmortem intervals were 0–41 h. The dataset had already been assembled into RPKM values based on ENSEMBL gene ID. Data is available at https://www.synapse.org/#!Synapse:syn3219045.

Further normalization and quality control procedures were applied to each of the datasets. First, outlier values for each gene were removed (standard deviation > 4). There were several samples for which many genes were outliers (> 300). These samples were removed. Overall, these samples had lower than average RIN scores. After removing those samples, the remaining outlier values were imputed with a K-nearest neighbor algorithm^40^. To put the datasets on a comparable scale, we scaled the dataset by mean (0-normalized) and standard deviation (normalized so the standard deviation for each gene is 1).

For continuous variables in ROS-MAP such as PD symptoms, we used a linear regression model subtracting *APOE ε4*, age, sex, race, and population principle components, study number, RNA integrity, and batch. Logistic regression was used in the case of binary variables such as PD diagnosis.

#### ROS-MAP characteristics

**Table:** 

Age range	67–108 years
Sex female	275 subjects
Average education	16.5 years
Race white	437 subjects
Race black	1 subject
Average post mortem interval	7.1 h
Past medical history of AD	170 subjects
Pathological diagnosis of AD	261 subjects
Past medical history of PD	31 subjects
Past medical history of Lewy body dementia	11 subjects
Self report history of thyroid problems	73 subjects
Self report history of heart problems	72 subjects
Self report history of stroke	43 subjects
Self report history of cancer	143 subjects
Self report history of hypertension	203 subjects

### Human brain tissue analysis

Brain tissue specimens were acquired from participants in the Religious Orders Study and provided by the Rush Alzheimer’s Disease Center, Rush University Medical Center in Chicago. Samples were taken from the mid-temporal cortex for every specimen. Samples were homogenized and protein was isolated as described in SDS-PAGE, Western.

Brain tissue specimens were also obtained from the Human Brain and Spinal Fluid Resource Center, VA West Los Angeles Healthcare Center, Los Angeles, CA 90073 which is sponsored by the NINDS/NIMH, National Multiple Sclerosis Society, and Department of Veteran Affairs (in collaboration with the NIH NeuroBioBank). Tissue samples were taken from the frontal cortex (coronal slab #4) for every specimen. Samples were homogenized and protein was isolated as described in SDS-PAGE, Western.

### Sodium-dodecyl-sulfate polyacrylamide gel electrophoresis (SDS-PAGE, Western) immunoblots

Tissues or cells were lysed in RIPA buffer (50 mM Tris at pH 7.4, 150 mM NaCl, 1 M EDTA, 0.25% deoxy chloric acid, and 1% NP-40) supplemented with protease inhibitor cocktail (Roche, Cat# 4693116001) and phosphatase inhibitors (20 mM sodium fluoride, 1 mM sodium orthovanadate). The mixture was centrifuged, and supernatant was taken. Protein levels were standardized using a Bradford protein assay. Protein was mixed with sodium dodecyl sulfate and electrophoresed in a 13% or 12% acrylamide gels. Proteins were transferred to a PVDF membrane (0.45 μM) and the membrane was then immunoblotted with antibodies, diluted at concentrations recommend by the manufacturer, against the specific proteins being examined. In this study we used the following Antibodies: anti-SIRT1 (rabbit polyclonal, gift from Imai Shin [47]), anti-SIRT6 (rabbit polyclonal, Sigma-Aldrich, Cat# S4322), anti-SIRT6 (rabbit monoclonal, Cell Signaling Technology, Cat# 12486, used only for Fig. 2f), anti-AKT (rabbit monoclonal, Cell Signaling Technology, Cat# 4691), anti-pAKT-S473 (rabbit monoclonal, Cell Signaling Technology, Cat# 4060), anti-Tyrosine Hydroxalase (rabbit polyclonal, Abcam, Cat# ab112), anti-NeuN (rabbit monoclonal, Abcam, Cat# ab177487), anti-TNF alpha (rabbit polyclonal, Abcam, Cat# ab9739), anti-β-actin (mouse monoclonal, Abcam, Cat#ab8226), anti-GFAP (mouse monoclonal, ThermoFisher Scientific, Cat# 50–9892). Band intensities were quantified using ImageJ.

### TNFα analysis

Commercial TNFα ELISA kits were used: Mouse TNFα DuoSet ELISA (R&D Systems, Cat# DY410). Media samples were analyzed in a Biotek Synergy 2 Multi-Mode Reader. Reads were normalized to cell number and amount of media present in the respective well.

### Proteasome activity assay

Protocol was followed from Proteasome Activity Fluorometric Assay Kit (BioVision, Cat# K245–100). Primary neuronal cultures were treated with nicotine 90 min before assessment. Samples were analyzed in a Biotek Synergy 2 Multi-Mode Reader.

### Real-time reverse polymerase chain reaction (RT-PCR)

Total RNA was extracted from tissues or cell culture using RNeasy kit (Qiagen, Cat# 74104). A cDNA library was prepared using Superscript III Synthesis System (Invitrogen, Cat# 18080051). Reverse polymerase reaction was performed using poly-dT primers as per manufacturers instruction. qRT-PCR was performed using CFX96 Touch™ Real-Time PCR Detection System, using the following primers: MAOB-F: ATGAGCAACAAAAGCCATGTCA; MAOB-R: TCCTAATTGTGTAAGTCCTGCCT; DAT1 F: AAATGCTCCGTGGGACCAATG; DAT1 R: GTCTCCCGCTCTTGAACCTC; VMAT2-F: AGGGGACACCTCTTACGACC; VMAT2-R: CTGCCACTTTCGGGAACACA; SIRT6-F: CTGAGAGACACCATTCTGGACT; SIRT6-R: GGTTGCAGGTTGACAATGACC; β-ACTIN-F: GACAGGATGCAGAAGGAGATCA; β-ACTIN-R: CTGATCCACATCTGCTGGAAGGT. All primers target mouse transcripts spanning exon junctions to eliminate DNA contribution to message quantification. All relative mRNA abundance measurements were to β-actin.

### MAO-B activity

MAO-B activity was analyzed by the commercial MAO-Glo™ Assay, Promega, Cat# V1401. Briefly, brain cortex homogenates were made in 100 mM HEPES 5% gylcerol buffer pH ~ 7.4. Homogenates were treated per MAO-Glo protocol and were treated with clorgyline (MAOA inhibitor, Abcam, Cat# ab145646) and or deprenyl (MAOB inhibitor, Abcam, Cat# ab120604). Samples were analyzed in a Biotek Synergy 2 Multi-Mode Reader.

### Animal experiments and transgenic mice

All procedures were performed according to guidelines and under supervision of the Institutional Animal Care and Use Committee (IACUC) of Cornell University. For all tests, we used both male and female 3-month-old mice. All transgenic animals were compared to corresponding wild-type littermates. Based on variability of prior collected data, necessary sample sizes were estimated using power analysis. No animals were excluded from the analysis; assignment to treatment groups was done at random using animal ear-tag numbers and done separately for males and females. All mice were handled daily for two weeks prior to the behavioral tests to eliminate the influence of stress and anxiety on their behavior due to human handling.

#### Conditional SIRT6 mice

The SIRT6 conditional deletion allele has been described previously [50]. To obtain brain-specific SIRT6 knockout animals (BSKO), we crossed these mice, which have exons 2 and 3 of SIRT6 flanked by loxP sites (Additional file 1: Figure S2 A-D), to a nestin-cre line of mice (JAX Stock# 003771).

To create conditional SIRT6 overexpressing mice (BSOX), chicken actin promoter (CAG) was cloned in front of chloramphenicol acetyltransferase (CAT) gene, flanked by loxP cites. Mouse SIRT6 cDNA with added polyA signaling sequence was cloned in after the second loxP site. This construct normally expresses reporter gene – CAT, upon exposure to Cre-recombinase, the CAT gene would be excised and SIRT6 will be expressed (Additional file 1: Figure S2 A-D). Linearized construct was injected into mouse embryo pronucleus, after which embryos were transferred into pseudo-pregnant dames. Pups were screened for the transgene presence by PCR and backcrossed into C57/BL for 8 generations. To overexpress SIRT6 specifically in the brain, resulting transgenic mice were crossed to nestin-cre line of mice (JAX Stock# 003771).

#### RNA sequencing and overrepresentation analysis

RNA was purified from dissected cortices of 3-month-old male BSKO, and BSOX mice, as well as their corresponding WT littermates (2 WT vs 2 BSKO, 2 WT vs 2 BSOX). Total purified RNA was depleted of cytoplasmic and mitochondrial rRNA using beads conjugated to oligonucleotides with sequences complimentary to those of ribosomal RNA. Purified and riboRNA-depleted RNA was fragmented and assessed for its quality and fragment size. cDNA library synthesis and adaptor/bar code ligation was done using Illumina TruSeq RNA Library Prep Kit, according to manufacturer instructions. The library was sequenced on an Illumina HiSeq instrument employing the 100 bp single-ended run regime. This configuration routinely resulted in ~ 200 million reads per run. Obtained reads were mapped onto *Mus musculus* genome using the open source software programs – Bowtie2 and TopHat. Expression of various loci (both coding RNA, and short and intragenic long non-coding RNA) was assessed across different genotypes using a related open source software program – Cufflinks and the R statistical package. Significantly altered genes were determined by *p*-values equal to or less than 0.05 after correction for false discovery. For overrepresentation analysis, genes significantly altered in BSKO and BSOX mice were combined and run with the Panther Classification System (http://www.pantherdb.org/). Separate analysis was performed for: Pathways, Biological Process, Cellular Component, and Molecular Function (Additional files 2, 3 and 4). *Mus musculus* was used for the gene reference list, and Bonferroni correction for multiple testing was performed.

#### MPTP

In vivo MPTP and nicotine mouse experiments: MPTP was delivered by intraperitoneal injection at 10 mg/kg four times a day for four days a week (two weeks’ total). Mice were scarified for immunohistochemistry 1 month after the completion of MPTP administration. Mice were 3 months of age at the start of treatment.

#### Nicotine

For in vivo administration, −(−) nicotine tartrate (MP Biomedicals, Cat# 0215355491) was added to tap water to create a concentration of free base nicotine at 200 μg per milliliter. Dilutions also contained 2% saccharin sodium salt hydrate (Oakwood Chemical, Cat# 098769). Control animals received only 2% saccharin. Nicotine dilutions were given in light protective bottles and made fresh 3 times a week. After 3 weeks of treatment mice were sacrificed and their tissues were analyzed. Mice were 3 months of age at the start of treatment.

### MPTP & nicotine cotreatment

Mice received nicotine and MPTP as described above, nicotine was given throughout the trial starting two weeks prior to MPTP injections. Mice were scarified for immunohistochemistry 1 month after the completion of MPTP administration. Animals were 3 months of age at the start of the trial.

### Open field

Behavior was measured in experimental animals two weeks after MPTP administration. In this period, animals were attended daily, to insure their wellbeing, as well as to habituate them to human handling to eliminate the influence of stress and anxiety on their behavior in the open field test paradigm. In this paradigm, a single animal was introduced to a novel square (90 cm by 90 cm), well-lit arena, cleaned of any familiar scents and filmed for 5 min. To take into consideration circadian changes in the behavior, all the experiments were performed at the same time of day, and no longer than 3 h in a row. Each mouse was recorded for at least five minutes. Mouse movement and statistics were analyzed automatically using custom-written tracking software, freely available upon request. At the time of data acquisition, identity of mouse genotype and treatment was not available to the person performing the analysis. Un-blinding was done only during statistical data analysis.

#### Rotarod

All mice were pre-trained on the rotarod apparatus to acclimate to the test. The pre-training consisted of four trials on 2 consecutive days, with an accelerating 4–40 rpm protocol reaching 40 rpm at 5 min. Latency to fall on the rotarod was recorded 1-week prior to sacrificing for immunohistochemistry (3-weeks after the start of MPTP regimen). Recording was done on two consecutive days with 3 trials for every mouse. The average of 6 trials for every mouse is presented.

#### Immunohistochemistry

Before brain sample collection, animals underwent cardiac perfusion with PBS for 5 min, and with 4% paraformaldehyde for 10 min. After perfusions, brain samples were quickly harvested and further fixed in 4% phosphate buffered paraformaldehyde solution for 16 h. After fixing, samples were cryo-protected in 30% sucrose for 30 h, embedded in OCT (Tissue-Tek) and stored at − 80 °C until use. Serial sections at 15 μm thickness were made on a cryostat and mounted on Superfrost Plus slides. Before staining, sections were dehydrated in acetone at 20 °C for 30 min, followed by three TBS washes before blocking in 1% normal goat serum for 2 h. Primary antibodies were diluted in TNT buffer (50 mM Tris at pH 7.5, 150 mM NaCl and 0.05% Tween 20; 1:500) and allowed for overnight incubation at 4 °C. Further staining steps were carried out with Vectastain Elite ABC HRP Kit (Vector Laboratories, now part of Maravai LifeSciences) according to the manufacturer’s instructions. Microscopic images were obtained using Aperio ScanScope SC2 system of total slide capture. Intensities of stain and cell counting were done using ImageJ software, macro functions were created for automatic quantification. Stereological assessment was also performed on tissue slides using Stereology Analyzer v 4.3.3 by ADCIS. A region of interest was outlined around *the substantia nigra pars compacta* on section slides, then a point grid was generated (100 × 100 pixels sampling and pattern, 1 pixel = 0.5 μm), if a grid point intersected a DA neuron it was counted, the volume fraction was subsequently computed. For each genotype/treatment, at least 5 sections from at least five mice were obtained and found to give a consistent pattern. Representative sections are shown in each case.

### Experimental statistical analysis

For pairwise comparisons, relevant to data analysis a two-tailed Student’s t-test was used and is usually reported on figures using “*”-representations, where *p < 0.05, **p < 0.01, ***p < 0.001, and ****p < 0.001. T-test was calculated assuming “equal variance” if variance of compared samples was similar. In cases where several conditions are being tested, one-way ANOVA was used, and performance and results of such analysis is described in figure captions. In cases were serval variables are influencing the same measured values, such as genotype and stress influencing survival of cells; two-way ANOVA analysis was performed. In this case three *p*-values are reported in figure legends, such as p-value for the influence of genotype on survival, p-value for the influence of drug on the survival and interaction p-value. Where appropriate, further post-hoc statistical tests were performed. Calculations were preformed using licensed and registered copy of Microsoft Excel or the open source free statistical software R, with Bioconductor package. For genotype association studies, a combination of R and p-link software was used, to create a linear regression model, where statistics were corrected for individual APOE ε4 status, age, sex, race, population principle components, RNA integrity, and batch as covariates. Bonferroni correction was used to account for multiple testing.

## Results

### Genetic variants and abundance of SIRT6 associate with TNFα and Parkinson’s disease in humans

**Fig. 1:**
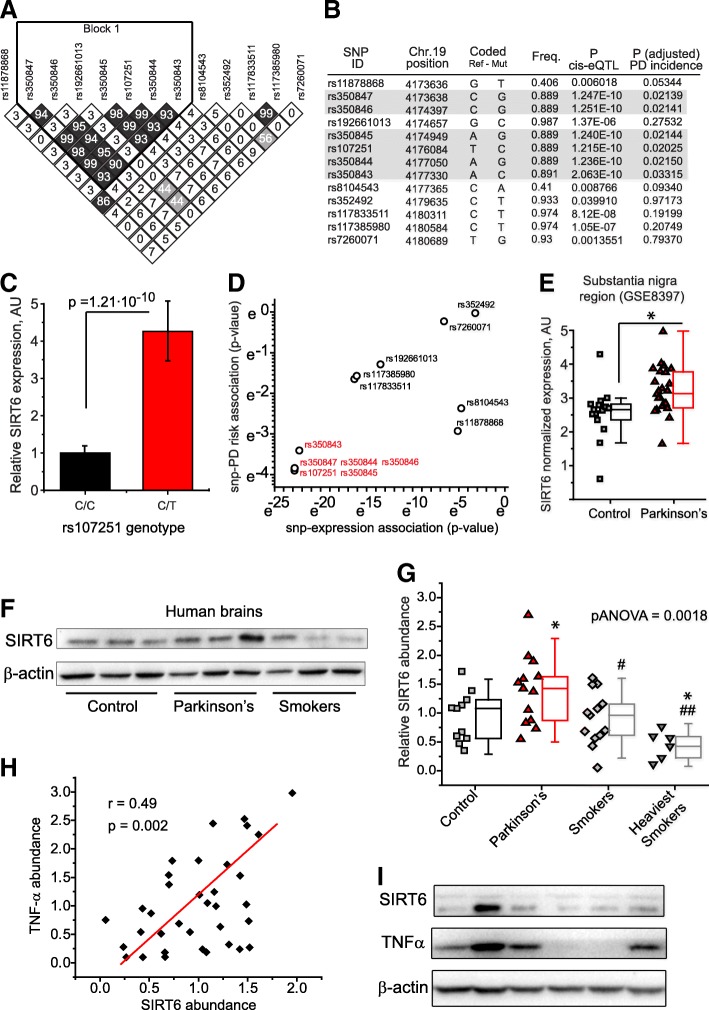
Higher expression of SIRT6 is associated with Parkinson’s Disease in humans. **a** Six N-terminus SNPs in SIRT6 are in strong linkage disequilibrium (LD). LD evaluation of genotyped SNPs in SIRT6 is shown. Shading of diamonds and numbers depict LD between markers based on the R^2^ measure, where a value of “44” corresponds to R^2^ = 0.44; (**b**) A summary of the associations between SNPs in SIRT6, SIRT6 gene expression, and Parkinson’s disease prevalence. SNP ID, position, major - minor alleles and frequency (Freq.), the nominal *p*-value for the association of the SNP with the SIRT6 expression, and the corrected p-value for the SNP association with PD prevalence are presented. We find that SNPs associated with elevated expression of SIRT6, also associate with increased prevalence of PD. The analyzed dataset is derived from ROS-MAP cohorts [6], in which all participants are organ donors. The dataset had already been assembled into RPKM values based on ENSEMBL gene ID and is publicly available through online Synapse archive (accession # syn3219045). **c** SNP rs107251 is associated with elevated expression of SIRT6 in human brains, bar graph showing the minor C/T genotype has ~ 4-fold greater expression of SIRT6 then major C/C. **d** The 13 SIRT6 SNPs analyzed from A/B, plotted by association with Parkinson’s Disease by effect on SIRT6 expression. Note that the SNPs that associate the most significantly with expression (red) also have the strongest association with Parkinson’s. **e** Box plot of the expression of SIRT6 in the substantia nigra region between control and PD patients, data is deposited to NCBI, accession number GSE8397. **f** Representative SDS-PAGE analysis of brain tissue lysates from healthy controls, Parkinson’s Disease patients, and tobacco smokers. See Additional file 1: Figure S1 for full blots. **g** Box plot quantification of SIRT6 protein levels (relative to β-actin), such as those presented on (F). * denotes significant difference compared from controls, # denotes significance from Parkinson’s patients. Student’s two tailed T-Test p-value for controls vs PD = 0.04, controls vs heavy smokers = 0.02, PD vs smokers = 0.02, PD vs heavy smokers = 0.002. One-way ANOVA between all groups p-value = 0.0018. See Additional file 1: Figure S1 for full blots used for quantification. Heavy smokers used two or more packs of cigarettes per day. **h** Scatter plot showing the correlation between SIRT6 and TNFα protein abundance. Pearson correlation = 0.49 and the slope of regression p-value = 0.002. See Additional file 1: Figure S1 for blots. **i** Representative western blot showing the positive correlation between the levels of SIRT6 and TNFα, such as those presented in (**h**)

To investigate the connection between SIRT6 and PD in humans, we performed a meta-analysis of published GWAS studies. First, we analyzed data from the Religious Orders Study (ROS) [6] and Rush Memory and Aging Project (MAP) [6] – ROS-MAP cohort, in which authors documented the medical history of participants, performed SNP genotyping, and measured genome-wide gene expression in the brain using RNA-sequencing. We analyzed thirteen SIRT6 SNPs reported in these studies and discovered that six SNPs, forming a linkage disequilibrium block in the N-terminus, have a significant impact (*p* < 10^− 9^) on the expression of SIRT6 (Fig. 1a-c). We tested the association of these SNPs with the incidence of PD and found that SNPs that associate with elevated expression of SIRT6 strongly associate with increased risk of PD (Fig. 1b, d). We verified that the identified SNPs associate with the risk of PD in five additional PD GWAS studies [41], which confirmed our original discovery. It is worth noting that the SNPs with the greatest impact on SIRT6 expression associate with PD incidence most strongly (Fig. 1d), suggesting a functional link. Moreover, reanalysis of published genome-wide expression data from the *substantia nigra* of healthy controls and patients with sporadic PD [38] revealed the latter tend to have elevated levels of SIRT6 transcripts (Fig. 1e).

Next, we acquired mid-cortex brain tissue samples from healthy controls, PD patients, and tobacco users [6]. After measuring SIRT6 abundance in these samples, we observed that SIRT6 protein levels are elevated in PD patient brains. Additionally, there is a negative correlation of SIRT6 abundance with tobacco use (Fig. 1f, g**,** Additional file 1: Figure S1). Those who smoked more than three packs of cigarettes a day had the lowest SIRT6 levels. Furthermore, we found that regardless of smoking or disease status, SIRT6 positively and significantly correlates with the abundance of the pro-inflammatory cytokine tumor necrosis factor alpha (TNFα) (Fig.1h, i**,** Additional file 1: Figure S1). These data suggest that elevated SIRT6 levels might increases the risk PD, and that tobacco use can suppress SIRT6 in human brain tissue.

### Tobacco and nicotine induce suppress SIRT6 in vitro and in vivo

**Fig. 2:**
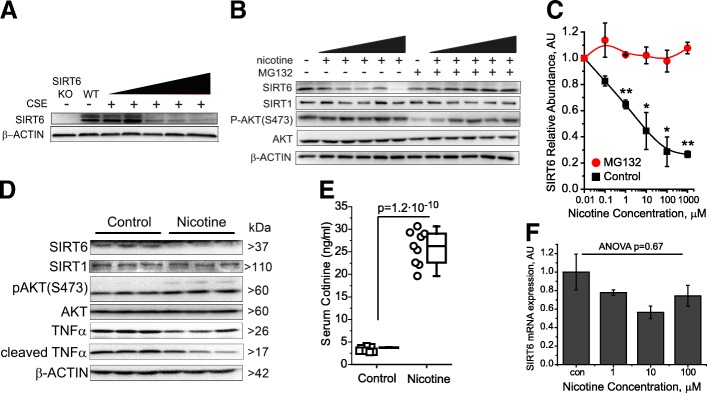
Nicotine suppresses SIRT6 in vitro and in vivo. **a** Typical SDS-PAGE analysis of primary murine neurons treated for 24 h with increasing concentrations of cigarette smoke extract (0.5, 1, 2, 5, and 10%). **b** Representative SDS-PAGE analysis of primary neurons treated for 90 min with nicotine at a range of concentrations - (0.1, 1, 10, 100, and 1000 μM); note the dose dependent decrease of SIRT6 abundance. Neurons pretreated with 10 μm MG132 were exposed for 2 h. **c** Quantification of SIRT6 protein levels from B, *n* = 3 independent experiments. Note a dose-dependent decline of SIRT6 protein levels in response to nicotine, and elimination of this effect by proteasome inhibition (mean ± SEM, one-way ANOVA: *p^nicotine^ = 0.008). **d** Representative SDS-PAGE analysis of brain lysates from animals treated with nicotine and vehicle-treated controls (same animals as shown in E). **e** Box plot of the concentration of serum cotinine in mice subjected to nicotine-supplemented water, *n* > 9 per treatment, two tailed t-test *p* < 10^− 9^. **f** Neurons treated with nicotine (1,10,100 μM) for 24 h, RNA was isolated and qRT-PCR was performed for SIRT6, two-tailed t-test performed between treatment and control groups. Note no significant difference in SIRT6 transcript levels (mean ± SEM, P-ANONA = 0.67)

Since both tobacco and SIRT6 expression are linked to PD, we tested if tobacco smoke influences SIRT6 abundance in neurons in vitro. To do so, we prepared cigarette smoke extract (Additional file 1: Figure S2) and treated primary murine neurons. We found that like in human smokers, the levels of SIRT6 can be decreased by tobacco in vitro (Fig. 2a). To test if nicotine itself reduces SIRT6 levels, we treated primary neurons with various doses of nicotine and found a dose-dependent decrease of SIRT6 abundance (Fig. 2b, c**,** Additional file 1: Figure S2C). The decrease of sirtuin levels by nicotine seems specific to SIRT6, as we observed no changes in the levels of the functionally similar SIRT1 (Fig. 2b). The reduction of SIRT6 in response to nicotine occurred rapidly, within 90 min of application, without changes in SIRT6 mRNA levels (Fig. 2f), all of which suggested a degradation mechanism. In support of this hypothesis, nicotine is known to regulate the ubiquitin-proteasome pathway in neurons [22], and SIRT6 has been shown to be regulated in a proteasome-dependent manner [31, 59]. To test this, we treated primary neurons with nicotine and the proteasome inhibitor MG132. We found that neurons with inhibited proteasome function do not decrease SIRT6 abundance in response to nicotine exposure (Fig. 2b, c**,** Additional file 1: Figure S2D). These data suggest that nicotine can accelerate proteasome-mediated degradation of SIRT6.

Next, we tested if nicotine in smoking-relevant concentrations can suppress SIRT6 abundance in vivo. To do so, we provided adult mice with drinking water supplemented with nicotine. We measured serum cotinine, an indicator of nicotine exposure, and found an average concentration of 26 ± 4 ng/mL in the nicotine-exposed mice (Fig. 2e), which is in the range of a typical human smoker [7]. Brain lysate analysis revealed that mice exposed to nicotine had ~ 50% reduction of SIRT6 levels (Fig. 2d), supporting our in vitro data. Importantly, both nicotine exposure and SIRT6 inhibition are known to activate AKT by phosphorylation at S473 [1], and we observed this in vitro and in vivo (Fig. 2b, d), supporting validity of our experiments. Taken together, these data demonstrate that nicotine in smoking-relevant concentrations can induce SIRT6 degradation in brain tissue in vivo.

### Loss of SIRT6 protects neurons from stress-induced apoptosis

**Fig. 3:**
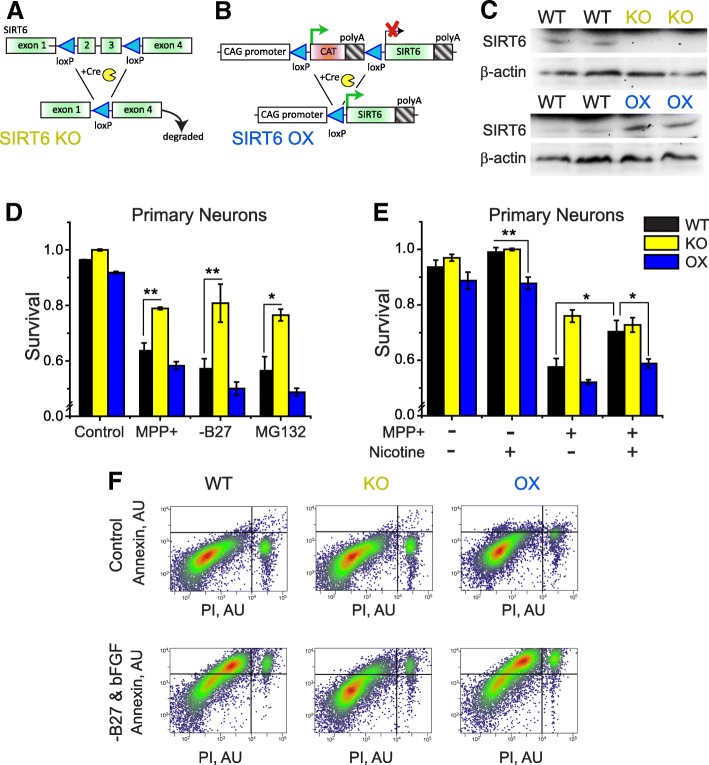
SIRT6 knockout neurons resist apoptosis and the effects of nicotine. **a** Schematic representation of the SIRT6 conditional strain of mice. Exons 2 and 3 (which comprise the enzymatic active site) of SIRT6 are flanked with *lox-P* sites, and when *cre-recombinase* is expressed, these exons are excised. **b** Schematic representation of SIRT6 conditional overexpressing (OX) transgenic allele. The chicken β-actin promoter is followed by a *lox-P* flanked stop site in all six reading frames, followed by SIRT6 gene cDNA and poly-A signal. For both KO and OX schemes, *cre-recombinase* is expressed from the *nestin* promoter, which allows creation of brain-specific SIRT6 knockout (BSKO) or brain-specific overexpression (BSOX) animals. **c** Typical western blot analysis of cortex lysates from BSKO or BSOX mice and their WT littermates. **d** Bar graphs representing surviving fraction of primary WT, KO, and OX neurons, cultured in vitro, 24 h after their stress with various insults. Neurons lacking SIRT6 resist apoptosis after stress. Con – control, MPP^+^ − 1-methyl-4-phenylpyridinium (500 μM), SS – starvation (in case of primary neurons, B27 serum and bFGF were withheld), MG – proteasome inhibitor MG132 (10 μM). Survival of cells was measured using flow cytometry, after cells had been stained with the apoptotic markers– Annexin-V and propidium iodide (PI). A cell was considered “alive” if it was negative for both Annexin-V and PI staining. Mean ± SEM, *n* = 3 independent experiments with at least 10,000 cells analyzed in each experiment for each treatment; two-tailed t-test analysis *p < 0.05, **p < 0.01, ***p < 0.001. **e** Survival of KO and OX neurons, respectively, pre-treated with 1 μM nicotine for two hours before MPP^+^ stress. WT cells pre-treated with nicotine had improved survival under stress, while SIRT6 KO and OX cells did not benefit further from nicotine pretreatment. **f** Representative flow cytometry plots showing WT, SIRT6 KO, and OX neurons starved and stained with Annexin-V and PI, included in analyses depicted in D. Each dot represents a single cell. Dot coloring reflects local cell density in the given area of the graph

To examine the causative relationship between SIRT6 and neuronal survival, we engineered brain-specific transgenic mice that either lack functional SIRT6 (BSKO, Fig. 3a), or overexpress SIRT6 by about four-fold (BSOX, Fig. 3b, c**,** Additional file 1: Figure S3A) specifically in the brain. Noteworthy, the magnitude of SIRT6 overexpression in the brains of BSOX mice is comparable to SIRT6 increase in humans with PD-associated SNPs (Additional file 1: Figure S3B). We isolated primary neurons from WT, BSKO, BSOX mice, and challenged them with a broad range of insults relevant to PD pathology. Following challenge, neurons were stained with the apoptotic markers Annexin-V and propidium iodide (PI), and their survival was measured using flow cytometry. Overall, SIRT6 KO neurons are better able to survive mitochondrial, oxidative, nutrient, and proteotoxic stress (Fig. 3d-f). Conversely, neurons that overexpress SIRT6 tend to have higher rates of apoptosis following these insults.

Nicotine is known to protect neurons from stress-induced apoptosis [35]. Given our preliminary data (Fig. 2), we hypothesized that the neuroprotective effect of nicotine might depend on SIRT6. To test this, we exposed WT neurons, and those with knocked out (KO) or overexpressed (OX) SIRT6 to nicotine and MPP^+^ (1-methyl-4-phenylpyridinium) - a molecule used to model PD-associated neuron death [64]. We found that after MPP^+^ challenge, nicotine improved survival of WT neurons; however, SIRT6 KO neurons did not receive protection from nicotine treatment (Fig. 3e). The neurons with enforced expression of SIRT6 had a marginal increase in survival after nicotine treatment (Fig. 3e), likely because nicotine-induced SIRT6 degradation was counteracted by the SIRT6 overexpressing transgene, dampening nicotine efficacy.

**Fig. 4:**
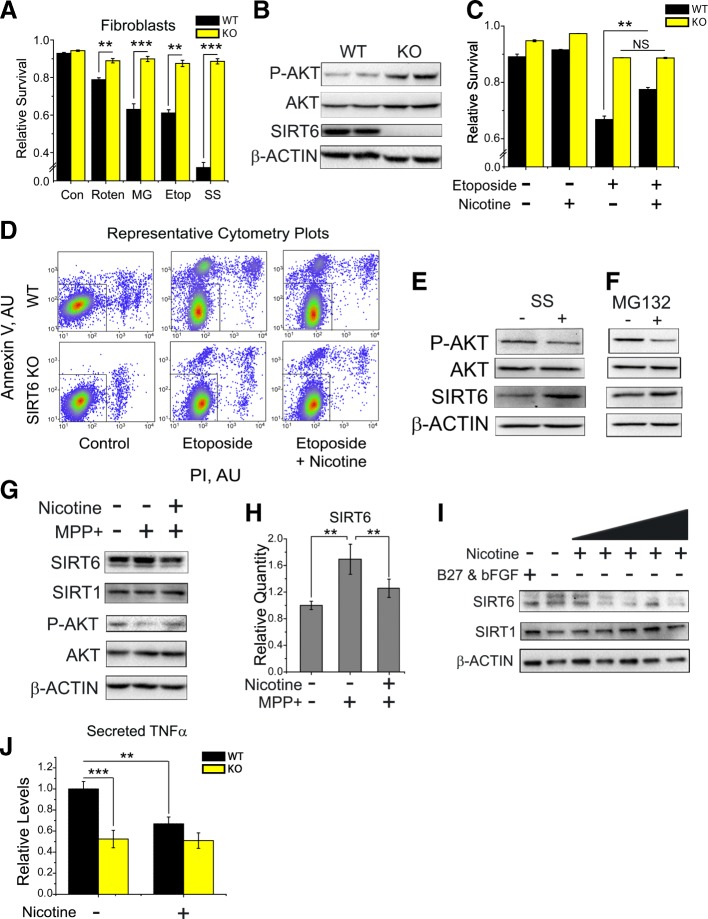
Nicotine reverses stress induced SIRT6 activity and inflammation. **a** Surviving fraction of WT and SIRT6 KO fibroblasts, cultured in vitro, 24 h after their stress with various insults. Fibroblasts lacking SIRT6 resist apoptosis after stress. Con – control, MPP^+^ − 1-methyl-4-phenylpyridinium (500 μM), SS – starvation (fetal bovine serum was withheld), MG – proteasome inhibitor MG132 (10 μM), Roten – rotenone (10 μM), Etop – etoposide (20 μM). Survival of fibroblasts was measured using flow cytometry, after cells had been stained with the apoptotic markers– Annexin-V and propidium iodide (PI). A cell was considered “alive” if it was negative for both Annexin-V and PI staining. Mean ± SEM, n = 3 independent experiments with at least 10,000 cells analyzed in each experiment for each treatment; two-tailed t-test analysis *p < 0.05, **p < 0.01, ***p < 0.001. **b** Typical western blot analysis of WT and SIRT6 KO fibroblasts. KO fibroblasts have higher levels of pAKT (S473) at baseline. **c** Survival of fibroblasts pre-treated with 1 μM nicotine for two hours before etoposide stress. WT cells pre-treated with nicotine had improved survival under stress, while SIRT6 KO cells did not benefit further from nicotine pretreatment. **d** Representative flow cytometry plots showing WT and SIRT6 KO fibroblasts stained with Annexin-V and PI, included in analyses depicted in C. Each dot represents a single cell. Dot coloring reflects local cell density in the given area of the graph. Survival of WT cells but not SIRT6 KO cells is improved by nicotine. **e** Typical western blot analysis of WT fibroblasts stressed with serum starvation. **f** Western blot analysis of WT fibroblasts stressed with MG132 (10 μM). SIRT6 increases under both SS and MG132 stress with a concomitant decrease in pAKT. **g** Typical western blot analysis of WT neurons treated with nicotine and or MPP^+^, as depicted in Fig.3e. Note the increase in SIRT6 from MPP^+^ stress and the lower levels under nicotine treatment. **h** Bar graph quantification of SIRT6 levels as depicted in G. **i** Typical western blot of WT neurons starved (of B27 and FGF) and treated with nicotine for 1.5 h (0.1, 1, 10, 100, and 1000 μM). SIRT6 increases after starvation but decreases upon nicotine exposure. **j** Bar graphs showing secretion of TNFα by primary neurons, measured by ELISA, 24 h after incubation with 1 μM nicotine. SIRT6 KO neurons secrete less TNFα than WT and are unaffected by nicotine. Mean ± SEM, *n* = 4 independent experiments, two-tailed T-test analysis for *, two-way ANOVA: p^genotype^ = 8.5•10^− 6^, p^nicotine^ = 8.8•10^− 3^, p^genotype x nicotine^ = 1.6•10^− 2^

We repeated the experiments testing the impact of SIRT6 and nicotine on cellular stress resistance in independently derived WT and SIRT6 KO fibroblast cell lines. As before, we found that SIRT6 KO fibroblasts have superior stress tolerance (Fig. 4a, b), and that nicotine protects cells from apoptosis in part through SIRT6 (Fig. 4c, d), confirming our initial observations.

SIRT6 activity has been associated with cellular stress before, and it was reported that SIRT6 abundance increases in cells after stress [36]. Our data is consistent with these reports. We find that after cellular stress, SIRT6 abundance is increased in both fibroblasts (Fig. 4e, f) and neurons (Fig. 4g, h). We also show that nicotine can reverse this stress-induced accumulation of SIRT6 and mitigate the downstream consequences (Fig. 4g, h, i, j), such as elevation of the pro-inflammatory cytokine TNFα. Taken together, these data demonstrate that the loss of SIRT6 enhances cellular survival under stress, and that nicotine at least partially functions through SIRT6 suppression to promote protection from apoptosis.

### SIRT6 regulates the pro-apoptotic TNFα pathway and pro-survival AKT signaling in the brain

**Fig. 5:**
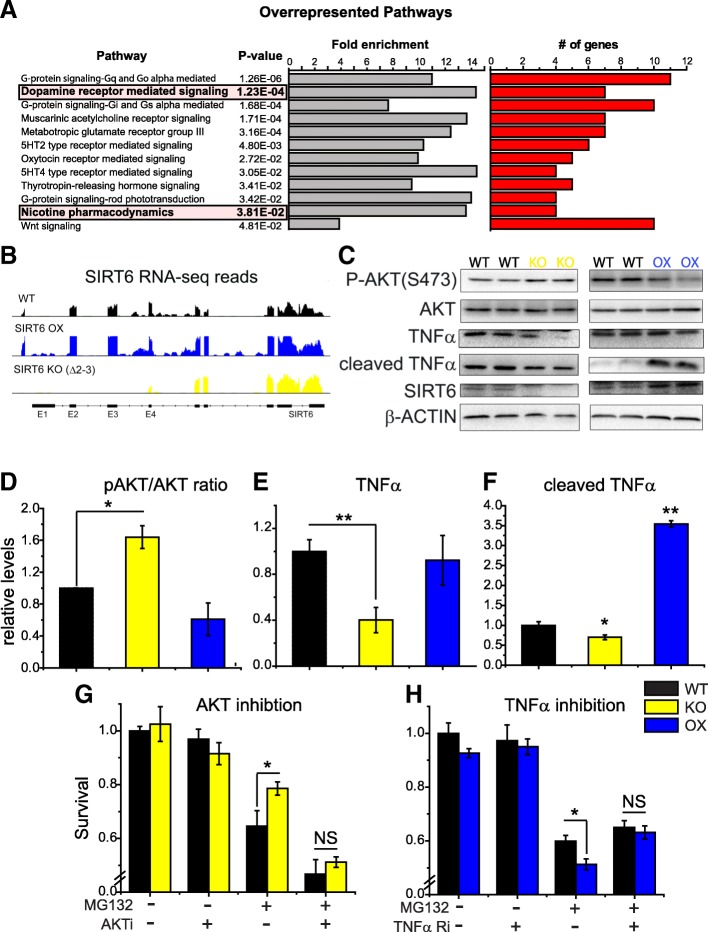
Characterization of brain-specific SIRT6 knockout and overexpressing mice. **a** Graphical representation of overrepresented pathways from RNA-sequencing analysis of BSKO, BSOX, and WT brains. All pathways shown were significantly altered after Bonferroni correction (*p* < 0.05). The number of genes affected from each pathway and the pathway fold enrichment is shown. See also Additional file 2 for complete data analysis. **b** Pile-up reads of SIRT6 from the RNA-seq analysis. BSKO mice lack reads for exons 2 and 3, while BSOX mice have increased reads at all exons. **c** Representative SDS-PAGE analysis of brain cortex lysates from BSKO, BSOX, and WT animals is shown. **d** Bar graph quantification of the ratio of phosphorylated (S473) AKT to total AKT from SDS-PAGE analysis, such as on c, showing a higher ratio (greater AKT activation) in BSKO brains (mean ± SEM, *n* ≥ 3, *p < 0.05). **e** Bar graph quantification of full TNFα, such as on C, show lower abundance of full length TNFα in KO brains (mean ± SEM, n ≥ 3, **p < 0.01). **f** Bar graph quantification of cleaved TNFα, such as on C, show lower abundance of cleaved TNFα in KO brains and greater abundance in OX brains (mean ± SEM, n ≥ 3, **p < 0.01). **g**, **h** Relative survival of WT, KO, and OX primary neurons assessed by PI/Annexin-V staining with AKT inhibitor (1 μm) or TNFα receptor inhibitor (100 ng/mL), and or 24 h of MG132 (10 μm) stress. (mean ± SEM, n ≥ 3, *p < 0.05)

We next explored the impact of SIRT6 dosage on brain physiology by profiling cortical gene expression by RNA-sequencing in BSKO, BSOX, and their respective WT littermates. Subsequent overrepresentation analysis showed that groups of genes involved in dopamine signaling and nicotine pharmacodynamics were significantly altered (Fig. 5a, b). These unbiased associations further strengthened SIRT6-nicotine-cell death connection. Another category impacted by SIRT6 was “immune-related processes” (Additional file 2). This is intriguing, since neuroinflammation and cytokines, namely TNFα, have been implicated in PD [2, 65]. Moreover, in vitro experiments have shown that SIRT6 regulates the production [62] and secretion [26] of TNFα. To investigate this link further, we measured the secretion of TNFα from primary neurons isolated from BSKO, BSOX, or WT brains. We found that SIRT6 KO cultures secrete less TNFα into the media (Fig. 4j), while OX cells secrete more than cells derived from WT littermates (Additional file 1: Figure S4A). Moreover, we found that nicotine suppresses TNFα secretion in WT cultures but does not affect it in KO neurons (Fig. 4j), which is consistent with a SIRT6-mediated action of nicotine. We also measured levels of TNFα in vivo and found that SIRT6 deletion leads to decreased levels of total and cleaved TNFα (Fig. 5c, e, f). Interestingly, we did not observe an upregulation of full TNFα in naïve BSOX mice, but rather significantly increased cleaved form, indicating greater secretion dynamics. Supportive of the link between nicotine and SIRT6, we observed a significant drop of TNFα (full and cleaved forms) in brains of mice treated with nicotine (Fig. 2d).

In addition to inflammation, we investigated AKT signaling in the brains of SIRT6 transgenic animals. The PI3K-AKT axis is a canonical pro-growth and pro-survival pathway [17], often disrupted in the brains of PD patients [20, 60]. Suppression of SIRT6 has been shown to increase AKT expression and signaling [51, 55, 56]. We found that AKT phosphorylation is increased in the brains of BSKO mice and KO fibroblasts (Fig. 5c, d**,** Fig. 4b), and is lower in brains of BSOX mice. To assess if AKT and TNFα play a role in SIRT6 mediated neuronal survival, we inhibited AKT activity or the TNFα receptor in primary KO and OX cultures respectively. We found that AKT inhibition blunted the pro-survival phenotype in KO cultures, while TNFα inhibition rescued the pro-apoptotic phenotype in OX cultures under stress (Fig. 5g, h). Overall, these data demonstrate that SIRT6 upregulates the pro-apoptotic TNFα pathway and suppresses pro-survival AKT signaling, which supports a pathogenic role for SIRT6 in PD.

### SIRT6 suppression confers neuroprotection in the MPTP model of Parkinson’s disease

**Fig. 6:**
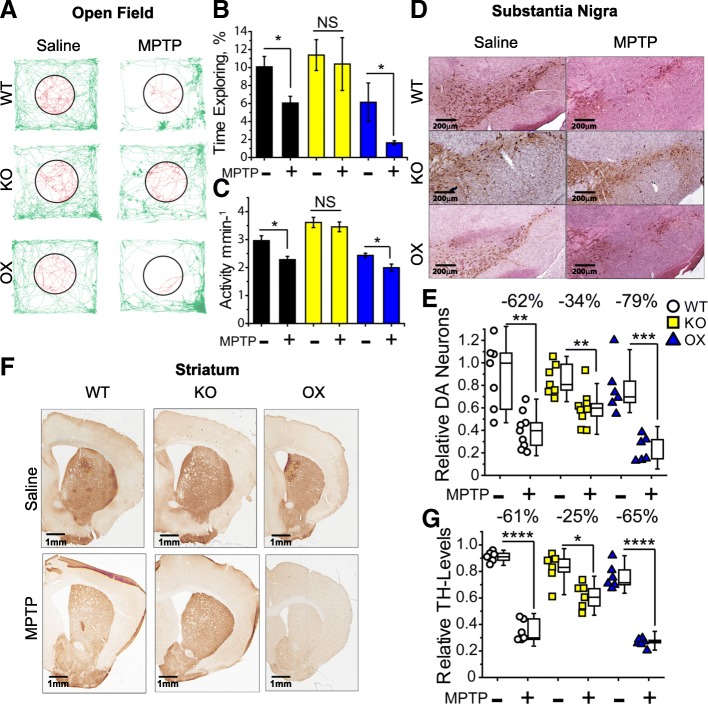
In vivo SIRT6 suppression protects from experimentally induced Parkinson-associated pathologies. **a** Representative movement traces from open field test of WT, BSKO, and BSOX mice treated with MPTP or saline. BSKO mice are more resistant to MPTP-induced motor dysfunction. **b** Mice are naturally afraid of open spaces and prefer to be next to a wall; the fraction of time the animal explores the center of the arena (red-shaded area in a is inversely related to the animal’s anxiety, which is induced in PD and by MPTP treatment. Quantification of exploratory behavior of SIRT6 transgenic mice treated with MPTP or saline is presented (Mean ± SEM, *n* ≥ 6, *p < 0.05 by two-tailed t-test. Two-way ANOVA: p^genotype^ = 6.6•10^− 5^, p^MPTP^ = 3.8•10^− 3^, p^MPTP x Gen^ = 4.8•10^− 2^). **c** Quantification of motor function of SIRT6 transgenic mice treated with MPTP or saline is presented via bar graphs (average distance covered per minute, mean ± SEM). BSKO animals are protected from PD-associated mobility decline (n ≥ 6, *p < 0.05 by two-tailed t-test. Two-way ANOVA: p^genotype^ = 4.8•10^− 8^, p^MPTP^ = 8.9•10^− 3^, p^MPTP x Gen^ = 0.675). **d** Representative immuno-histochemical analysis of *substantia nigra pars compacta* of WT, BSKO, and BSOX mice treated with MPTP or saline. 15 μM sections were stained with anti-Tyrosine Hydroxylase to visualize dopaminergic neurons (brown stain) and counterstained with hematoxylin (purple stain). BSKO animals demonstrate reduced reduction of DA neurons after MPTP treatment, and BSOX animals have exaggerated neuronal death. **e** Quantification of histochemical analysis presented on d. Boxplots illustrate mean values and standard deviation of the relative abundance of DA neurons; box whiskers represent 5th and 95th percentiles of data distribution. Each circle is a separate animal. Black boxes – WT, yellow – BSKOs, and blue – BSOX animals (n ≥ 6, **p < 0.01, and ***p < 0.001 by two-tailed t-test, two-way ANOVA: p^genotype^ = 6.2•10^− 2^, p^MPTP^ = 8.2•10^− 11^, p^MPTP x Gen^ = 4.4•10^− 3^). **f** Representative immuno-histochemical analysis of *striatum* of WT, BSKO, and BSOX mice treated with MPTP or saline. DA neuron projections are visualized with anti-TH antibody. Drop in the density of dopaminergic projections after MPTP injections is mitigated in BSKO animals, and is exaggerated in BSOX mice. **g** Quantification of histochemical analysis, such as those presented on (**f**). Boxplots illustrate the mean density of dopaminergic projections in *striatum* of experimental animals; the box structure and coloring is the same as in e, (*n* ≥ 6, *p < 0.05, and ****p < 0.0001 by two-tailed t-test, two-way ANOVA: p^genotype^ = 2.3•10^− 6^, p^MPTP^ = 3.5•10^− 16^, p^MPTP x Gen^ = 1.1•10^− 5^)

To investigate the impact of SIRT6 on neurodegeneration in vivo, we utilized our brain-specific SIRT6 transgenic mice and the MPTP-based model of PD. MPTP (1-methyl-4-phenyl-1,2,3,6-tetrahydropyridine) selectively induces death of DA neurons and produces pathologies that closely mimic human PD; these include: neuroinflammation, nigrostriatal damage, changes in behavior and physical activity [37]. After BSKO, BSOX, and WT littermates were treated with MPTP, we assessed their behavior and activity using an open field test. Compared to WT littermates, BSKO animals were protected from physical activity decline (Fig. 6a-c) and anxiety increase (Fig. 6a, b) induced by MPTP. Subsequent immuno-histochemical analysis demonstrated that BSKO mice suffered less nigrostriatal damage: they had significantly more surviving DA neurons in the *substantia nigra* (Fig. 6d-e) and greater DA neuron dendrite density in the *striatum* relative to WT (Fig. 6f-g). Conversely, BSOX mice showed exaggerated PD-like symptoms– they had increased susceptibility to behavioral changes and elevated DA neuron death compared to WT littermates.

**Fig. 7:**
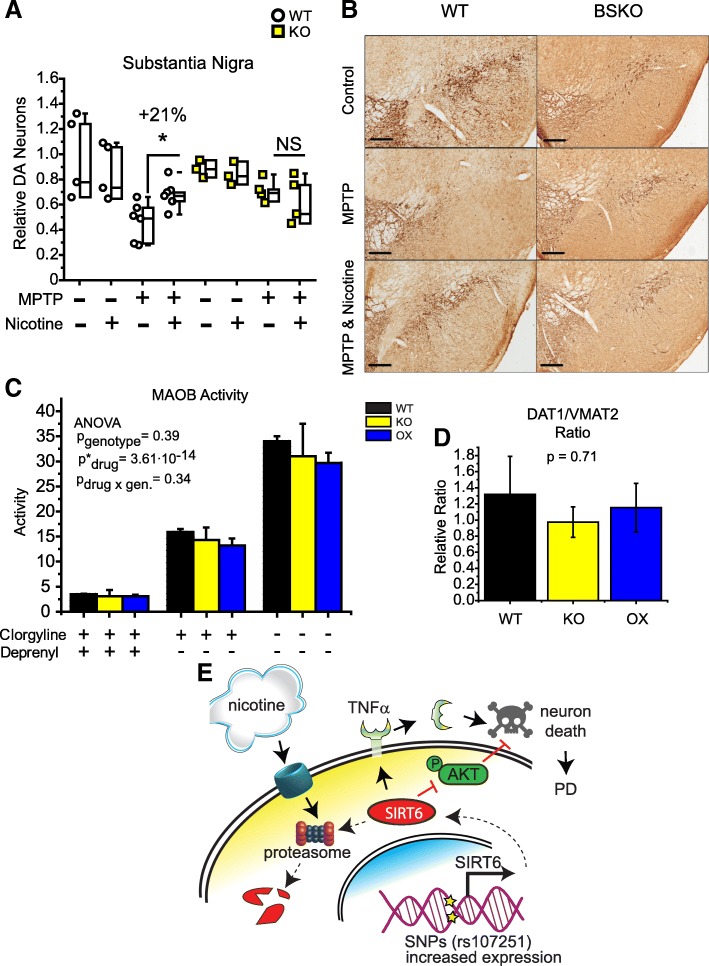
Nicotine does not rescue MPTP-induced DA neuron death in SIRT6 brain-specific knockout mice. **a** Quantification of histochemical analysis presented on (**b**). Boxplots display the relative density of DA neurons in the *substantia nigra pars compacta* assessed by stereological counting. Three-way ANOVA analysis: p^genotype^ = 0.57, p^MPTP^ < 0.0001, p^nicotine^ = 1.0. (**b**) Representative immuno-histochemical analysis of *substantia nigra pars compacta* of WT and BSKO mice treated with MPTP and or nicotine. Stained with TH to visualize DA neurons. (**c**) Bar graph of MAOB enzymatic activity from cortex homogenates. Total MAO activity was not different; residual MAOB activity was not different when MAOA was inhibited with clorgiline; nor residual MAOA activity was different when MAOB was inhibited with deprenyl. Mean ± SEM, *N* ≥ 3, p^genotype^ = 0.39, p^inhibitors^ = 3.61•10^− 14^, p^genotype x inhibitors^ = 0.34 by two-way ANOVA). Together these data suggest that SIRT6 does not alter the metabolism of the neurotoxin MPTP. **d** Relative DAT1/VMAT2 ratio calculated from individual expression data from WT, BSKO, and BSOX brains. See also Additional file 1: Figure S3. One-way ANOVA *p* value shown. **e** A schematic of the mechanistic link between nicotine, SIRT6, and PD is illustrated. Our data demonstrates that SIRT6 plays a pathogenic role in initiation and progression of PD by stimulating TNFα release and suppressing AKT signaling, all of which promote neuronal apoptosis. SNPs associated with an increased risk of PD, such as rs107251 significantly increase SIRT6 abundance and therefore increase likelihood of neuronal apoptosis. Nicotine promotes proteasome-dependent degradation of SIRT6, which in turn protects cells from stress-induced apoptosis and prevents or ameliorates neurodegenerative pathologies. Selective targeting of SIRT6 might have a therapeutic effect against PD

To examine the relationship between SIRT6, nicotine, and neuroprotection in vivo, we challenged WT and BSKO mice with MPTP, while simultaneously co-treating them with nicotine. WT mice treated with nicotine received partial protection from MPTP-induced DA neuron death, while BSKO mice did not benefit from nicotine (Fig. 7a, b). Additionally, we performed a motor assessment before sacrificing the mice (Additional file 1: Figure S5), which corroborates the partial protection afforded by nicotine. These data support our in vitro findings (Figs. 3d and 4c) and suggest that SIRT6 inhibition partially mediates nicotine’s neuroprotective action.

The neurotoxicity of MPTP depends on the activity of monoamine oxidase B (MAOB), vesicular monoamine transporter 2 (VMAT2), and the dopamine transporter 1 (DAT1) [46]. We measured the expression of MAOB, VMAT2, and DAT1 and enzymatic activity of MAOB in the brains of BSKO, BSOX, and WT mice. We found that the DAT1/VMAT2 ratio and enzymatic activity of MAOB were unaltered by SIRT6 dosage (Fig. 7c, d). The expression of MAOB, VMAT2, and DAT1 were also not significantly different between WT and transgenic mice, corroborating the RNA-seq expression results (Additional file 1: Figure S3C-E, Additional files 3 and 4). These data suggest that BSKO animals resist MPTP-induced damage via enhanced DA neuron survival and not by alterations in MPTP metabolism.

## Discussion

Our study provides a new molecular link between nicotine, PD risk, and SIRT6. We suggest that SIRT6 is a plausible therapeutic target against PD. As far as we understand, genetic predisposition or cellular stresses that result in SIRT6 induction promote neuroinflammation and cell death (Fig. 7e), accelerating neurodegeneration. Nicotine or other molecules that can inhibit SIRT6 activity or prevent SIRT6 accumulation might enhance neuronal survival under stress (Fig. 3). We find that both SIRT6 isoforms are sensitive to nicotine and tobacco extract. In addition, it was previously found that the lower molecular weight isoform of SIRT6 is the predominate isoform in neuronal cultures [52], which we also typically observed. Our data demonstrates that nicotine can suppress SIRT6, likely through proteasome-mediated degradation, which is intriguing since nicotine has been shown to suppress proteasome activity [44]. We postulate that nicotine can suppress overall proteasome activity (Additional file 1: Figure S2E) but simultaneously target and degrade certain proteins, such as SIRT6. The mechanism of this redirection is currently unknown and might be an interesting topic for future studies.

The pro-survival kinase AKT is reported to regulate SIRT6 degradation [59]; however, our experiments suggest an alternative mechanism, since we do not observe a correlation between AKT activity and the degree of SIRT6 reduction (Fig. 2b). Our data also support the conclusion that AKT signaling itself is regulated by SIRT6 activity.

It is fascinating that tobacco use reduces the prevalence of PD, while almost universally being detrimental to other diseases. Tobacco smoke consists of thousands of compounds, some of which might have very strong anti-PD properties. We and others suggest that nicotine is one of these molecules. Nicotine has been shown to have beneficial effects in animal models of PD and clinical trials have been encouraging, but more work is needed to determine the proper administration and efficacy [32, 48, 58]. Future studies might identify additional tobacco components that regulate sirtuins, neuronal survival, and neurodegeneration. These findings support the therapeutic potential of SIRT6 in PD.

In agreement with previous reports [26, 55, 62], we find that suppression of SIRT6 increases AKT signaling and reduces the secretion of TNFα, both of which likely mediate the impact of SIRT6 on DA neuron survival and PD pathology (Figs. 5g, h and 7e). Our data show that nicotine can reduce the abundance and secretion of TNFα in a SIRT6 dependent manner. SIRT6 levels also positively correlate with TNFα abundance in human brain tissue, further supporting an inflammatory and pathogenic role for SIRT6 in PD and corroborating a connection between SIRT6 and TNFα (Fig. 1h).

Previous studies have also demonstrated a pro-apoptotic role for neuronal SIRT6 in culture [9, 43], supporting our results. We find that four-fold overexpression of SIRT6 is sufficient to alter gene expression and enhance MPTP-induced pathology and neuron death in vivo (Fig. 6). Human SNPs that associate with a similar four-fold increase in SIRT6 expression significantly elevate PD risk (Fig. 1a-d). Additionally, brain tissue from PD patients have elevated levels of SIRT6 protein (Fig. 1f, g**,** Additional file 1: Figure S1), further supporting a PD-SIRT6 association.

Recent studies have reported that SIRT6 protects from DNA-damage associated with Alzheimer’s disease (AD) [28, 29]; more specifically, Kaluski et al. showed that cells without SIRT6 succumbed to apoptosis faster, following DNA-damage induced by ionizing radiation. We find SIRT6 KO cells (fibroblast lines and primary neurons) are more resistant to apoptosis induced by oxidative damage, proteotoxicity, and nutrient shortage, which is supported by other independent studies [51, 63, 67] and our previous work [14]. It is possible that the effect of SIRT6 on stress-induced survival depends on the nature of the stress. It is also possible that there is a fundamental difference between AD and PD pathogenesis and stress, which lends SIRT6 as protective in one and pathogenic in the other. On this note, it is intriguing that DA neurons were shown to have extremely high levels of NAD+ [61], which could make these cells more sensitive to SIRT6 induced cell death.

Several studies propose that SIRT6 is an attractive target for activation, as it is shown to suppress survival of cancer cells [25, 59] and extend longevity in whole body overexpressing mice [30]. Our data support the role of SIRT6 in the regulation of cell death, but add caution to potential therapies promoting its activity, because it may exacerbate death of DA neurons (among other cell types) and accelerate PD-associated degeneration. In the same regard, the implementation of inhibition therapies to promote cellular or neuronal survival must consider a potential carcinogenic pitfall. While there are many studies detailing the positive effects of sirtuin activity in aging and disease states, our data suggests that (at least for SIRT6) the outcome is context, cell-type, and disease dependent.

Finally, it should be noted that SIRT6 has at least three reported enzymatic activities: deacetylation [55], de-fatty acylation [26], and ADP-ribosylation [36]. A compelling topic for future studies would be to investigate if any of these activities have a dominant impact on SIRT6’s role in neuronal and cellular survival, as well as to investigate the efficacy of transient SIRT6 suppression on PD (mimicking a clinical therapy). A detailed analysis of the relationship between SIRT6 and nicotine’s receptors and associated neuroprotective pathways should also be carried out [54] (Additional file 5). Such experiments will inform the development of activity-specific SIRT6 inhibitors that could be used for the treatment of PD.

## Conclusions

The reduced prevalence of Parkinson’s disease in tobacco users is a fascinating phenomenon that is not understood. This study suggests a mechanistic explanation for how tobacco users are protected from Parkinson’s and how the tobacco component nicotine confers neuroprotection; more specifically, nicotine suppresses SIRT6 which confers resistance to neuron and cell death. Few effective treatments exist that prevent neuron death for those suffering from Parkinson’s and other neurodegenerative disorders. The identification of SIRT6 as potentially pathogenic and as a therapeutic target for suppression opens a novel line of research for the treatment of neurodegeneration.

## Additional files

Additional file 1: **Figure S1.** Human brain tissue analysis by SDS-PAGE. **Figure S2.** Cigarette smoke extract apparatus, nicotine blots, and proteasome activity. **Figure S3.** Transgenic mice and brain expression. **Figure S4.** SIRT6 OX neurons secrete more TNFα than WT. **Figure S5.** Primary neuronal culture composition. **Figure S6.** Nicotine does not rescue MPTP-induced rotarod motor performance in SIRT6 brain-specific knockout mice. (DOCX 2045 kb)

Additional file 2: RNAseq overrepresentation analysis. (XLSX 32 kb)

Additional file 3: RNAseq WT vs BSOX mice. (XLSX 2739 kb)

Additional file 4: RNAseq WT vs BSKO mice. (XLSX 2649 kb)

Additional file 5: nAChRs and SIRT6 interaction analysis. (XLSX 274 kb)

## Abbreviations

BSKO: Brain-specific SIRT6 knockout
BSOX: Brain-specific SIRT6 overexpressing
DA: Dopaminergic
KO: Knockout
MPTP: 1-methyl-4-phenyl-1,2,3,6-tetrahydropyridine
OX: Overexpressing
PD: Parkinson’s Disease
MPP +: 1-methyl-4-phenylpyridinium
TH: Tyrosine Hydroxylase
