# Supplementary material for: Nicotine promotes neuron survival and partially protects from Parkinson’s disease by suppressing SIRT6
Source: Acta Neuropathol Commun. 2018 Nov 8;6:120. doi: 10.1186/s40478-018-0625-y (PMC6223043; doi:10.1186/s40478-018-0625-y)
Supplement: Supplementary file 1 — Figure S1. Human brain tissue analysis by SDS-PAGE. Figure S2. Cigarette smoke extract apparatus, nicotine blots, and proteasome activity. Figure S3. Transgenic mice and brain expression. Figure S4. SIRT6 OX neurons secrete more TNFα than WT. Figure S5. Primary neuronal culture composition. Figure S6. Nicotine does not rescue MPTP-induced rotarod motor performance in SIRT6 brain-specific knockout mice. (DOCX 2045 kb) [file 40478_2018_625_MOESM1_ESM.docx]

**SUPPLEMENTARY INFORMATION**


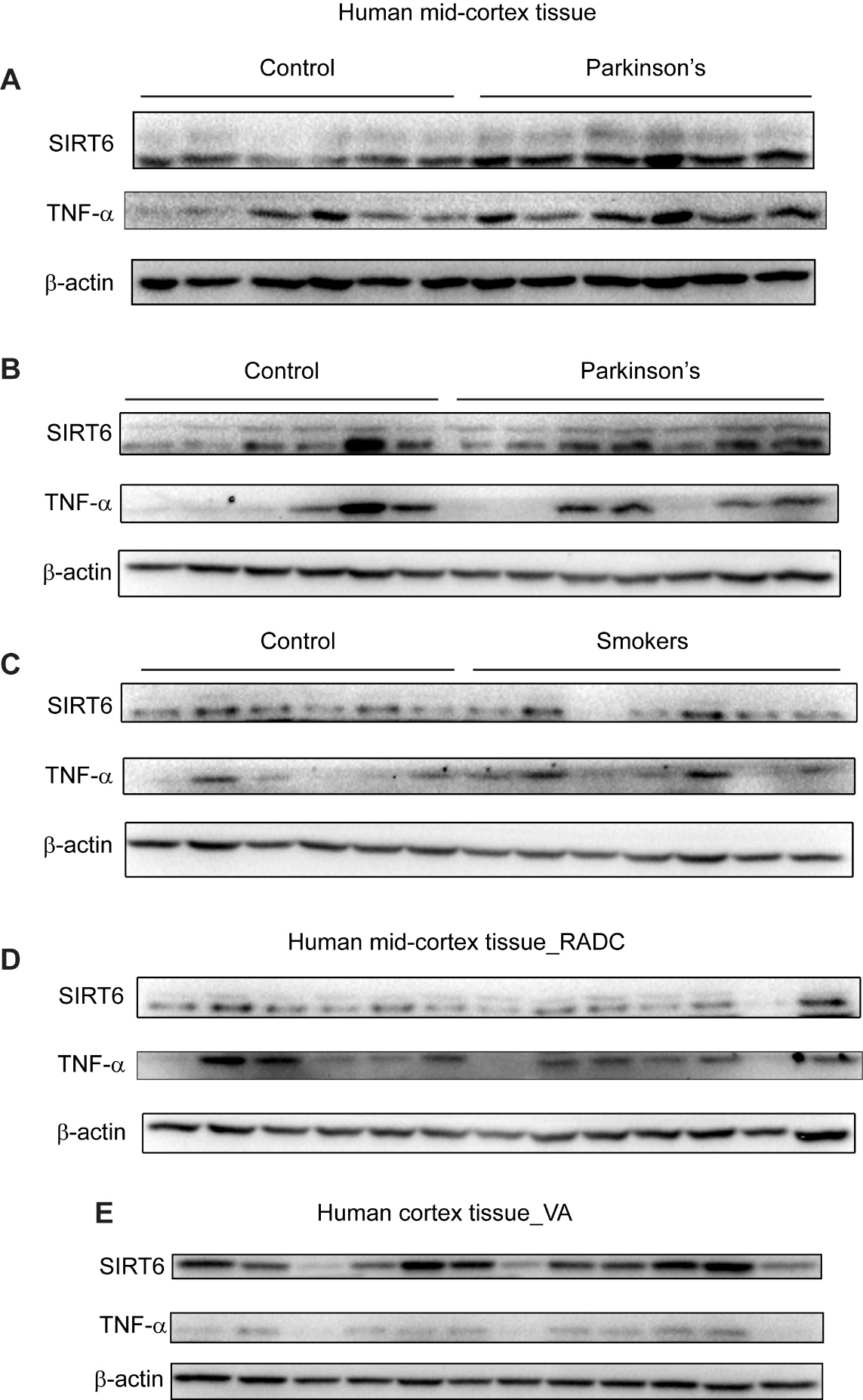
­­

**Figure S1. Human brain tissue analysis by SDS-PAGE.**

**(A-E)** Western blot analysis of human brain tissues showing abundance of SIRT6, TNFα, and β-actin. Each lane is an individual bio-specimen. Blots were used for quantifications showed in Figure 1G and H. RADC (Rush Alzheimer’s Disease Center), VA (Veteran’s Affairs), See Methods for details of brain tissue analysis.


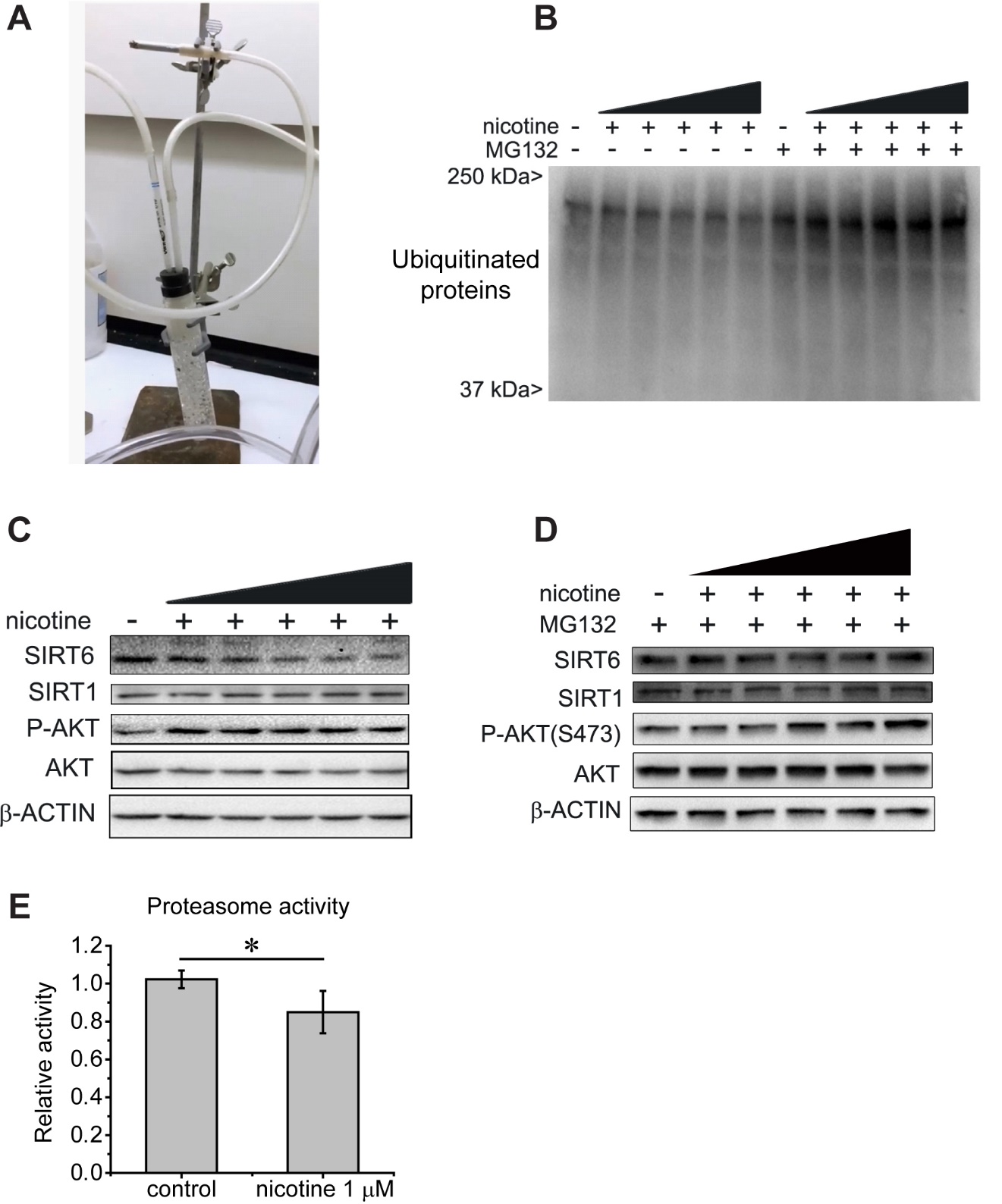


**Figure S2. Cigarette smoke extract apparatus, nicotine blots, and proteasome activity.**

**(A)** Picture of cigarette smoke extract apparatus used in our studies and for data presented on Figure 2A.

**(B)** SDS-PAGE analysis of samples as shown in Figure 2B blotted for ubiquitin. Note the increase in ubiquitinated proteins with the proteasome inhibitor MG132.

**(C)** Representative SDS-PAGE analysis of primary neurons treated for 90 minutes with nicotine at a range of concentrations - (0.1, 1, 10, 100, and 1000 μM); note the dose dependent decrease of SIRT6 abundance.

**(D)** Representative SDS-PAGE analysis of primary neurons treated for 90 minutes with nicotine at a range of concentrations - (0.1, 1, 10, 100, and 1000 μM), and pretreated with MG132 for 2 hours.

**(E)** Overall proteasome activity of primary neuronal cultures treated with nicotine as in C.


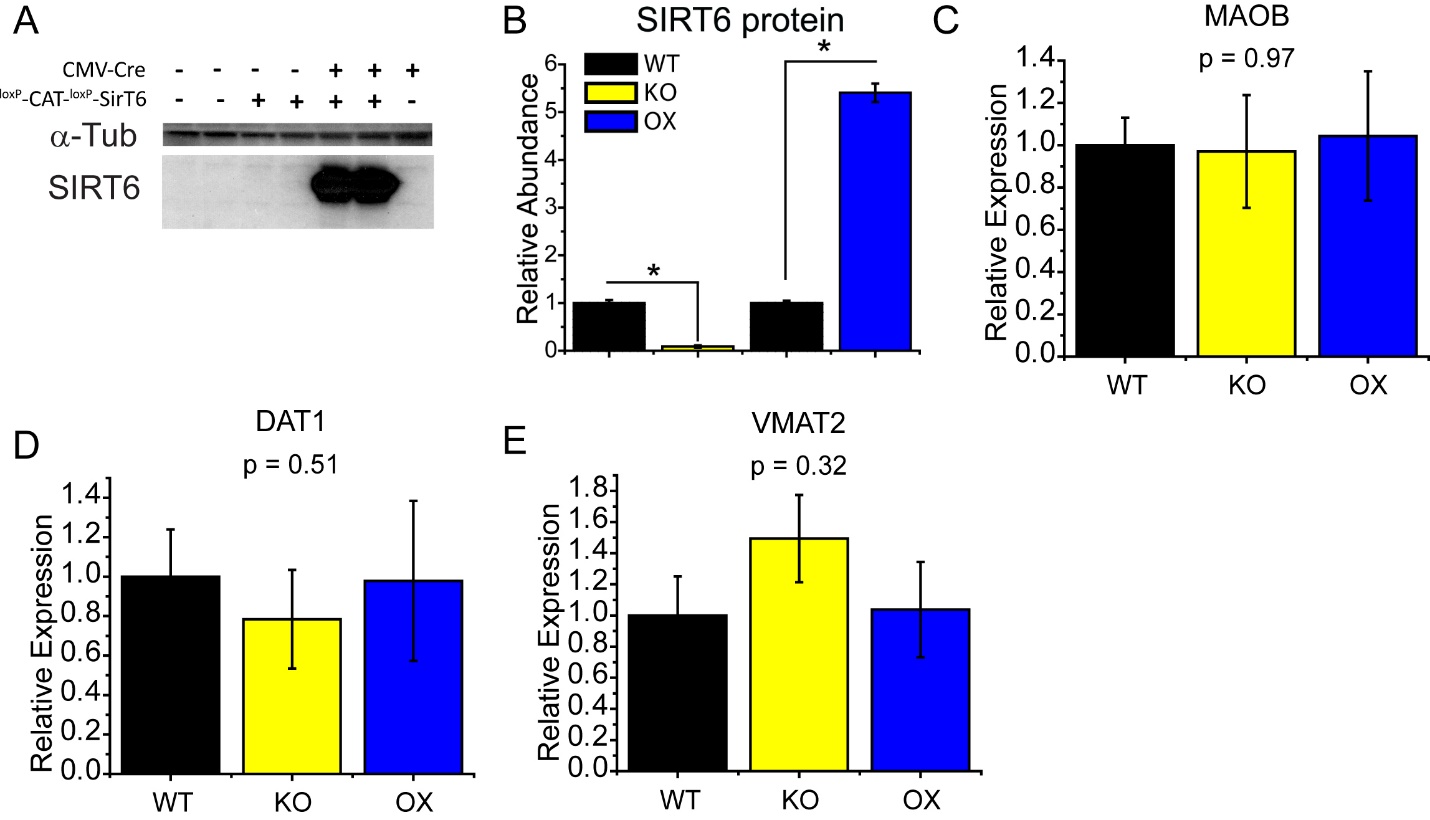


**Figure S3. Transgenic mice and brain expression.**

**(A)** Typical western blot analysis of SIRT6 overexpressing transgenic cassette, which was used in creation of BSOX mice. Note that cassette does not normally express SIRT6, however in presence of *Cre*-recpmbinase, when STOP-signal is excised, overexpression ensues.

**(B)** Bar graph of quantification of SIRT6 protein from brain lysates of the transgenic mice (WT, BSKO, and BSOX) (mean±SEM, N=4, *p<0.05 by two-tailed t-test).

**(C, D, E)** Expression of MAOB, DAT1, and VMAT2 from the brains of WT, BSKO, and BSOX mice quantified by qRT-PCR. P value of one-way ANOVA shown (n = 8), note no significant effect from genotype.

**
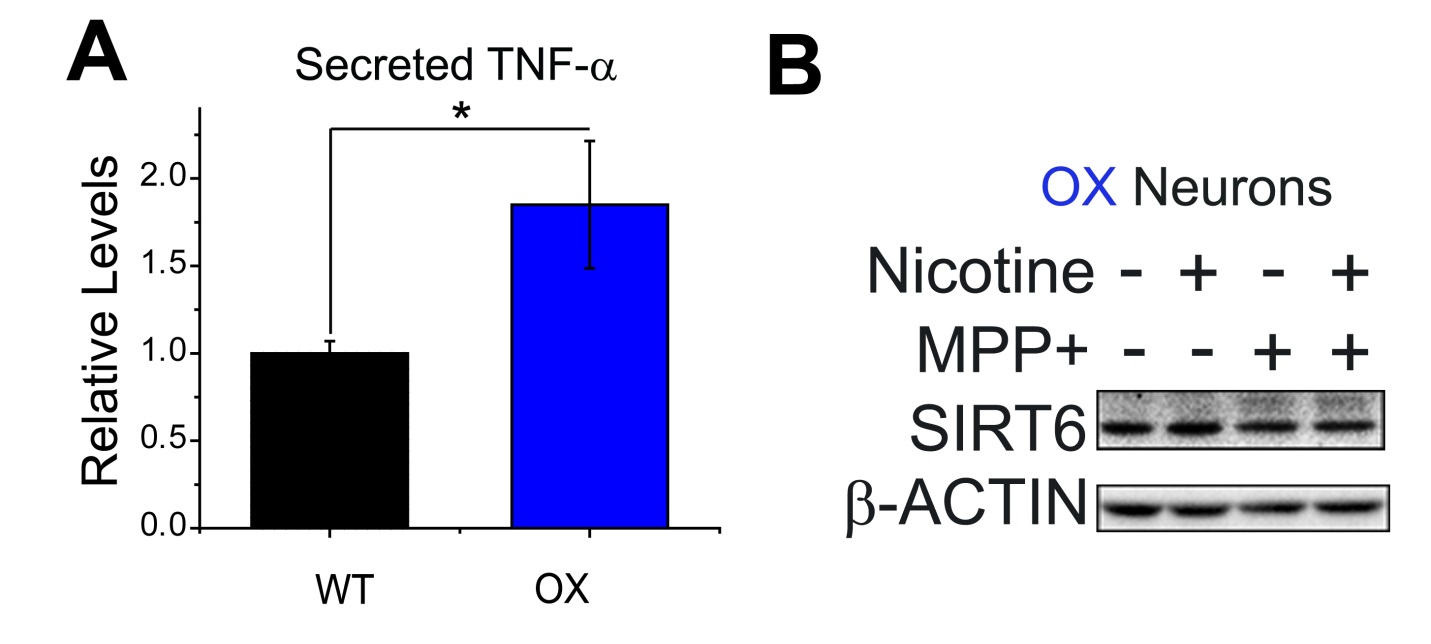
**

**Figure S4. SIRT6 OX neurons secrete more TNF**α **than WT.
(A)** Bar graph showing secretion of TNFα by primary SIRT6 overexpressing neurons, measured by ELISA. At baseline, SIRT6 OX neurons secrete more TNFα than their WT counterparts (mean±SEM, n=3 independent experiments, two-tailed t-test analysis shown, one-way ANOVA: p^genotype^=0.003).

**(B)** Representative western blot analysis of SIRT6 OX neurons stressed with MPP^+^ (500 μM) for 24 hours with and without nicotine (1 μM) pre-treatment, as depicted in **Fig. 3E**. SIRT6 increases upon MPP^+^ stress and remains elevated when nicotine is present.


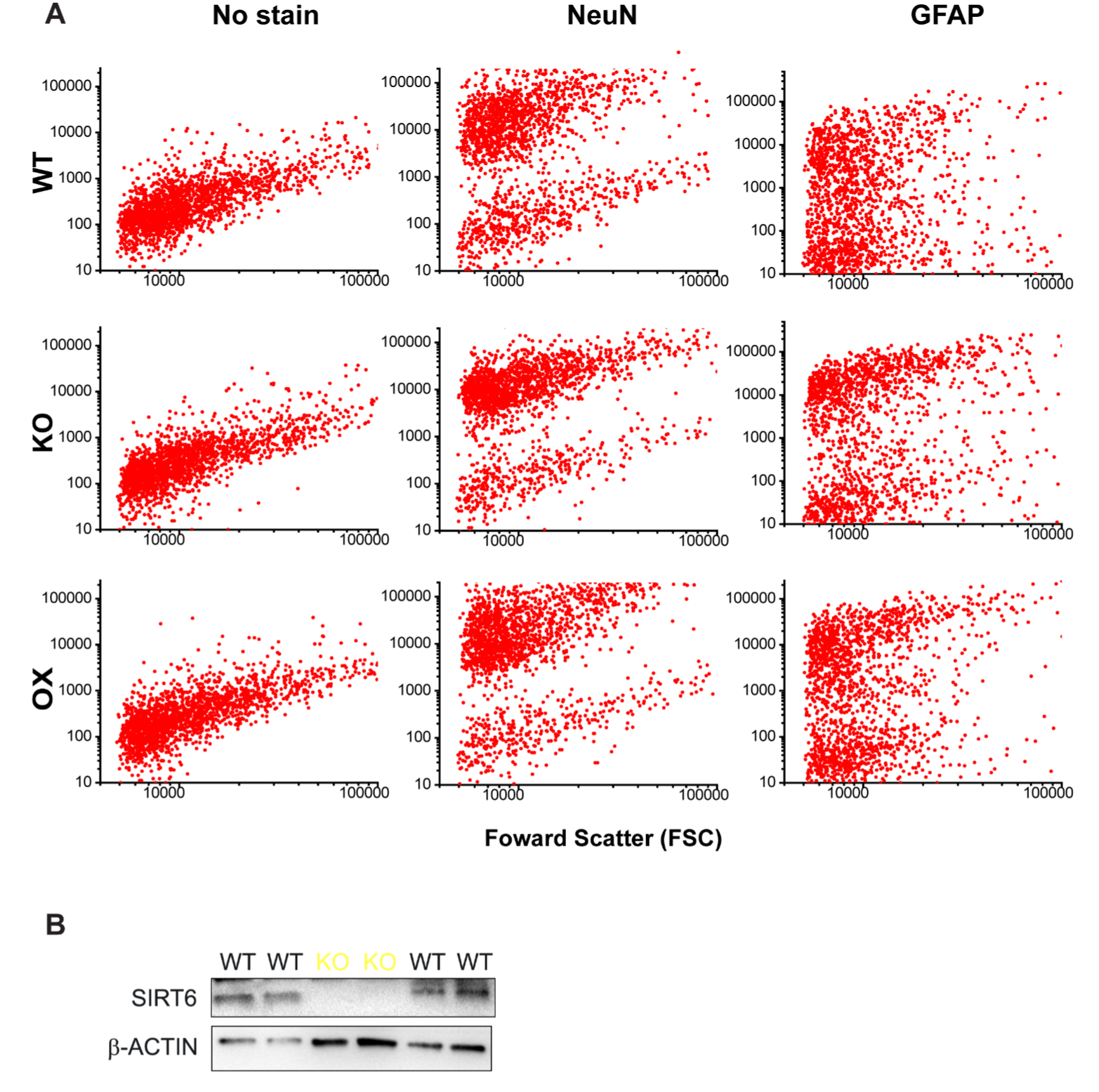
 **Figure S5. Primary neuronal culture composition.**

**(A)** Representative flow cytometry plots of WT, SIRT6 KO, and OX neuronal cultures stained with NeuN (neurons), GFAP (astrocytes), or unstained. Note that all cultures proportions of neurons and astrocytes are equivalent and that cells stained with NeuN constitute the majority.

**(B)** Western blot analysis of WT and KO primary neuronal cultures. Note the complete absence of SIRT6 in KO lanes, even when overloaded for total protein compared to WT. This indicates the absence of microglia in these cultures since few if any neonatal microglia express nestin, which is the driver of CRE in our transgenic model.


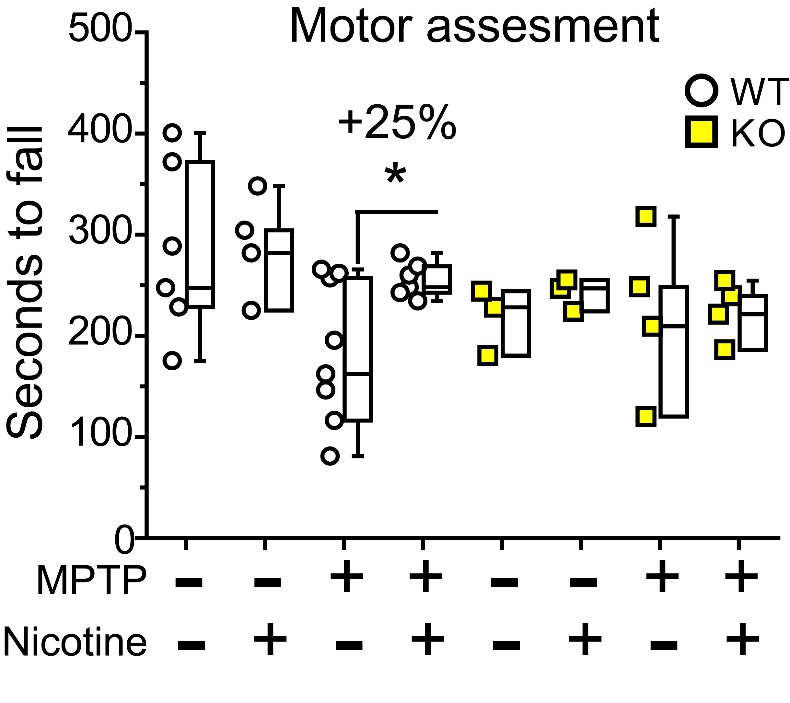


**Figure S6. Nicotine does not rescue MPTP-induced rotarod motor performance in SIRT6 brain-specific knockout mice.**

Boxplots display the latency until fall (in seconds) from the rotarod performance test with forced motor activity (see methods for details). Three-way ANOVA analysis: p^genotype^=0.33, p^MPTP^=0.02, p^nicotine^=0.17
